# Electrocardiographic Discrimination of Long QT Syndrome Genotypes: A Comparative Analysis and Machine Learning Approach

**DOI:** 10.3390/s25072253

**Published:** 2025-04-02

**Authors:** Martina Srutova, Vaclav Kremen, Lenka Lhotska

**Affiliations:** 1 Department of Natural Sciences, Faculty of Biomedical Engineering, Czech Technical University in Prague, 272 01 Kladno, Czech Republic; 2 Czech Institute of Informatics, Robotics and Cybernetics, Czech Technical University in Prague, 160 00 Prague, Czech Republic; vaclav.kremen@cvut.cz

**Keywords:** long QT syndrome, LQT3 discrimination, electrocardiogram parameterization, electrocardiogram classification, support vector machine classification

## Abstract

Long QT syndrome (LQTS) presents a group of inheritable channelopathies with prolonged ventricular repolarization, leading to syncope, ventricular tachycardia, and sudden death. Differentiating LQTS genotypes is crucial for targeted management and treatment, yet conventional genetic testing remains costly and time-consuming. This study aims to improve the distinction between LQTS genotypes, particularly LQT3, through a novel electrocardiogram (ECG)-based approach. Patients with LQT3 are at elevated risk due to arrhythmia triggers associated with rest and sleep. Employing a database of genotyped long QT syndrome E-HOL-03-0480-013 ECG signals, we introduced two innovative parameterization techniques—area under the ECG curve and wave transformation into the unit circle—to classify LQT3 against LQT1 and LQT2 genotypes. Our methodology utilized single-lead ECG data with a 200 Hz sampling frequency. The support vector machine (SVM) model demonstrated the ability to discriminate LQT3 with a recall of 90% and a precision of 81%, achieving an F1-score of 0.85. This parameterization offers a potential substitute for genetic testing and is practical for low frequencies. These single-lead ECG data could enhance smartwatches’ functionality and similar cardiovascular monitoring applications. The results underscore the viability of ECG morphology-based genotype classification, promising a significant step towards streamlined diagnosis and improved patient care in LQTS.

## 1. Introduction

Long QT syndrome (LQTS) is an inherited cardiac condition characterized by delayed ventricular repolarization, a critical electrophysiological substrate predisposing individuals to life-threatening arrhythmias. With an estimated prevalence of 1 in 2000 to 2500 [1], this condition exhibits variable expressivity and incomplete penetrance, influenced by genetic, environmental, and physiological factors. The pathophysiological hallmark of LQTS is the prolongation of the QT interval on the electrocardiogram (ECG), arising from defects in ion channel genes responsible for cardiac action potential propagation. Among the genotypes, LQT1, LQT2, and LQT3 predominate, each with distinct triggers and molecular underpinnings affecting potassium and sodium ion channel kinetics [2].

The dire clinical manifestations of LQTS, including syncope, torsades de pointes (TdP), and sudden cardiac death, particularly in the young, underscore the importance of precise genotype identification [3]. Yet, the gold standard for diagnosis—genetic testing—remains limited by accessibility, cost, and time constraints [2,4]. Non-genetic triggers specific to genotypes, such as exercise in LQT1, auditory stimuli in LQT2, and rest and sleep states in LQT3, compound the complexity of clinical management and risk stratification [4,5,6].

LQT3 patients are at higher risk due to their inability to easily avoid trigger activities that are unavoidable in life. Moreover, pharmacological treatment with beta-blockers, which are the first intervention choice and are protective in LQT1 and LQT2, offers only suboptimal protection [1,7,8], or even worsening of the condition, when they may facilitate TdP [9] in carriers of the LQT3 genotype. Recognizing these challenges, our research aims to leverage signal processing and machine learning to enhance the discrimination of LQTS genotypes via ECG signal analysis.

The diagnostic role of T-wave morphology biomarkers in LQTS is well-described and comprehensively summarized in the systematic review by Tardo et al. [10]. Most current studies addressing LQTS primarily focus on distinguishing LQTS patients from non-affected individuals and those with borderline QT intervals. Building on the foundation of these studies, we introduce advanced algorithmic strategies aimed at refining genotype differentiation further.

There are only a few studies that directly focus on the genotyping of LQTS patients, with most attention given to the LQT1 and LQT2 groups. Despite the serious implications for patients with the LQT3 genotype, this group has not received sufficient attention. The differentiation of the LQT3 genotype is mentioned briefly in the publication [11], which reports an 80% accuracy for LQT3 using a classifier based on a convolutional neural network model.

The primary contribution of this work is the introduction of novel geometric approaches to parameterization. The proposed parameters are grounded in previously conducted modeling and in vitro studies [12,13,14], which describe ECG manifestations depending on the genotype and the severity of the disease.

Another practical study, from which the parameter proposal is derived, addresses experimental observations of ST-T patterns. These patterns are documented in the research work published by Zhang et al. [15]. This study also highlights a general limitation of this work: a partial overlap of ECG patterns for LQT1 and LQT3, which affects our results as well. This work seeks to push the boundaries in addressing these limitations.

The parameters proposed in this study are not only specifically applicable to LQTS genotyping but, we believe, can have broader applications. The ECG-derived features, based on the transformation of waveforms into unitary geometrical spaces, are highly transparent and demonstrate low computational complexity. The applicability of these parameters has been validated by their use in the SVM classification model, leveraging only single-lead ECG data with a 200 Hz sampling frequency. This demonstrates their potential for enhancing smartwatches’ functionality and similar cardiovascular monitoring applications.

Such a machine learning approach promises to accelerate the identification of at-risk individuals, guide therapeutic decisions, and pave the way for personalized medicine in the management of LQTS, all with minimal costs.

## 2. Materials and Methods

### 2.1. Data Acquisition and Study Population

This investigation employs a robust Holter ECG Warehouse (THEW) dataset, specifically the genotyped long QT syndrome subset E-HOL-03-0480-013 [16]. This comprehensive repository furnishes extensive ECG recordings alongside genetic profiles of individuals diagnosed with LQTS. These longitudinal ECG datasets have been meticulously curated to provide a high-resolution temporal window into the cardiac rhythms of patients with genetically confirmed LQTS, offering an unparalleled opportunity for in-depth analysis of the electrophysiological signatures characteristic of distinct LQTS genotypes.

The data compilation comprises a diverse cohort of subjects with a confirmed diagnosis of LQTS, ensuring a broad representation of the genotypic spectrum within the condition. Each subject’s dataset encompasses a substantial sequence of ECG tracings, securely anonymized in compliance with patient confidentiality protocols. These tracings have been recorded with rigorous adherence to quality standards, thus providing a dependable foundation for subsequent computational analyses [16].

#### 2.1.1. Inclusion Criteria

To qualify for inclusion in this study, participants’ ECG records were required to demonstrate precise genotypic categorization as either LQT1, LQT2, or LQT3 based on genetic testing results embedded within the database. Records were also screened to meet quality control metrics, including clarity of signal, absence of excessive noise, and the presence of complete data for the entire recording duration.

#### 2.1.2. Data Processing and Selection

The raw ECG data underwent preprocessing, focusing on discrete segments of the ECG recordings corresponding to periods of wakefulness between 10 a.m. and 8 p.m., presuming regular diurnal variations in cardiac function. This strategic selection criterion aims to minimize the confounding influences of circadian rhythm variations on the QT interval, thereby enhancing the precision of the genotype discrimination process.

This study’s rigorous data selection and preprocessing protocols establish a high-quality, genetically validated dataset primed for applying machine learning techniques to discriminate between LQTS genotypes with enhanced accuracy and clinical relevance.

#### 2.1.3. Dataset Characterization and Preparation

The dataset for this study was meticulously constructed following the aforementioned criteria, culminating in a robust assembly of ECG signals. The dataset was based on one of the largest available LQTS databases [16], which includes 246 gender balanced records validated as the LQT1 genotype, 145 as the LQT2 genotype, and 35 as the LQT3 genotype. The time duration of signals is in the range of approximately 17–24 h for each patient. Specifically, the dataset comprises a collection of ‘resting vigilance’ ECG traces—signals captured when patients are awake but at rest—predominantly from Lead I, renowned for its diagnostic value in LQTS detection. The fidelity of these signals is preserved at a sampling frequency of 200 Hz, which is deemed sufficient to capture the nuances of QT interval variances while facilitating computational efficiency. Each ECG trace in the dataset spans 60 cardiac beats, striking a balance between providing adequate data for analysis and maintaining manageability for algorithmic processing.

The dataset is stratified into three groups, corresponding to the LQT1, LQT2, and LQT3 genotypes. The number of ECG signals representative of each genotype is denoted as 205 for LQT1, 86 for LQT2, and 31 for LQT3, respectively (Table 1). This stratification allows for targeted analysis and comparative evaluation among the different LQTS genotypes.

#### 2.1.4. Control Group Selection

To establish a baseline for testing and validation (Table 1), a subset comprising one-third of the ECG signals from each genotype group was sequestered to form a control cohort. This control group was selected through a randomized process to ensure unbiased representation across the genotype spectrum. The randomized selection process not only strengthens the robustness of the control group but also mirrors the stochastic nature of the clinical presentation and enhances the generalizability of the study’s findings.

These control sets, representative of each LQTS genotype, were utilized exclusively for the testing phase of the machine learning model. Delineating a distinct testing cohort is crucial for evaluating the model’s discriminative performance. When confronted with previously unseen data, it ensures that the results reflect the algorithm’s true predictive capabilities.

In conclusion, the preparation and delineation of the dataset form the cornerstone of this research, providing a solid foundation upon which the comparative analysis and machine learning models can be developed and evaluated. The dataset’s thoughtful compilation and rigorous preparation underscore the meticulous approach undertaken to ensure the integrity and reliability of the subsequent analysis.

### 2.2. Extraction of Signal Parts

**Figure 1:**
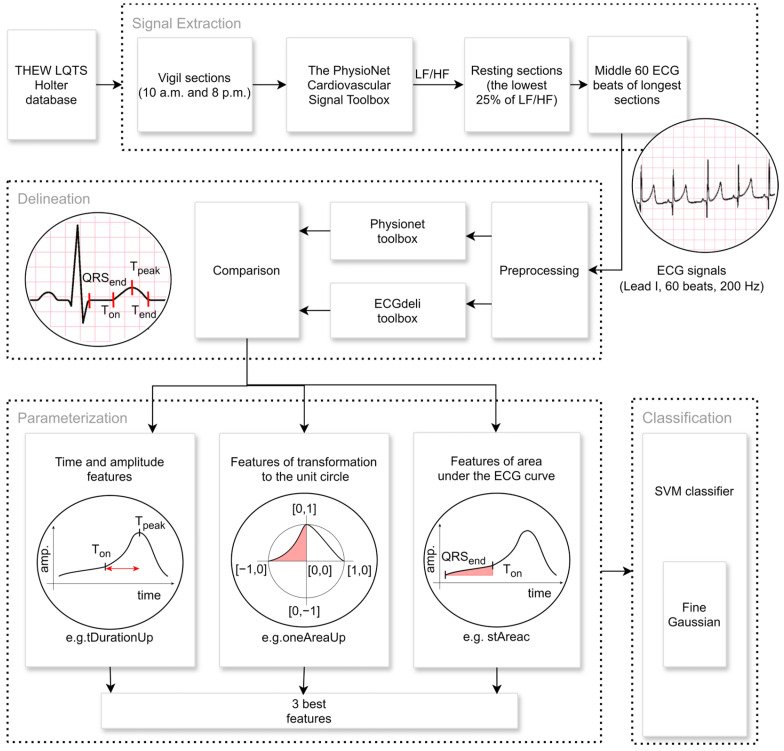
Signal extraction, preprocessing, and feature extraction pipeline: The extraction part includes the selection of the ECG signals from the period between 10 a.m. and 8 p.m. when we assume that the patient is in a state of vigilance. The resting part of the size of 60 ECG beats is selected, whereby it is estimated from the LF/HF heart rate variability parameter [17]. The part of such a signal is visualized in the first circle picture. Two freely available algorithms are used, the Physionet toolbox [18] and ECGdeli [20], and the crucial points of each ECG are specified in the delineation part. Finally, the triad of the best mutually uncorrelated parameters utilizes the novel approaches of the calculation of the area under the curve or the transformation to the unit circle, in addition to traditional methods. The appropriate features (*tDurationUp*, *oneAreaUp*, and *stAreac*) for LQT3 differentiation are visualized in the last three circle pictures. They are chosen as inputs for the SVM classification model to differentiate the LQT3 genotype.

Only parts of the signals from the time between 10 a.m. and 8 p.m. are chosen when vigilance/awakening of the patient is assumed. Twenty-five percent of this signal with the lowest heart rate variability (HRV) parameter LF/HF, the ratio of the power in the low and high frequency range [17], which mirrors the sympathovagal balance, is declared to be at rest. The LF/HF is successively calculated by the PhysioNet Cardiovascular Signal Toolbox [18,19] in sections of 200 beats in length with an offset of 100 beats. The 60 ECG beats in the middle of the two previously described most extended vigilance resting sections are subsequently preprocessed and delineated by the Physionet [18] and ECGdeli [20] toolboxes. Primarily, the Physionet delineation is applied. If it fails, the ECGdeli delineation is used instead. Subsequently, the signals are partially displayed, and each patient’s best is manually selected. In addition, noisy signals or signals where the delineation is unsuccessful are excluded. All calculation processes (Figure 1) are designed and implemented in the MATLAB program environment, as well as the aforementioned toolboxes.

### 2.3. Feature Extraction

The chosen parts of each signal are described using the parameters (A) traditionally used to analyze ECG signals. Subsequently, we suggest two new approaches that present a new dimension to the perspective of ECG signal parameterization. (B) The calculation of the area under the ECG curve describes the combination of the duration and amplitude effect. Against it, the attitude of (C) wave transformation into the unit circle and its parametrization suppress the influence of the time and amplitude of the ECG signal. The primary objective of this method is to quantify the shape of individual ECG beat components, with a particular emphasis on characterizing the concavity and convexity of these segments. The analysis focuses predominantly on the ST segment and the T-wave area.

**Figure 2:**
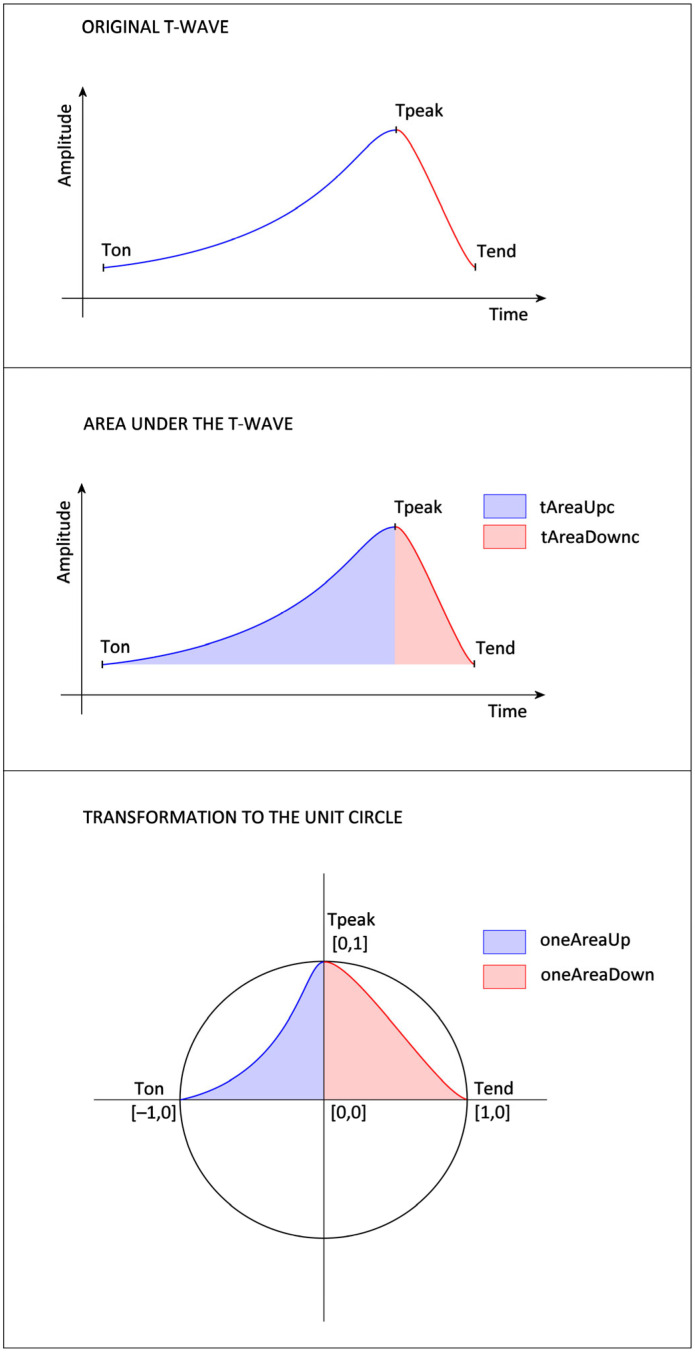
Proposed feature extraction from the ascending (blue) and descending (red) parts of the T-wave (**top**). The approach includes calculating the area under the T-wave curve with parameters denoted as *tAreaUpc* (blue) and *tAreaDownc* (red) (**center**), the transformation to the unit circle (**bottom**), and the values of features *oneAreaUp* (blue) and *oneAreaDown* (red) extracted from this transformed T-wave to the unit circle.

A. Time and amplitude features extracted from ECG:

- *tDuration*: The time duration between the T-wave’s beginning (T_on_) and the end (T_end_) (Figure 2).
- *tDurationUp*: The time duration between the T-wave’s beginning (T_on_) and peak (T_peak_) (Figure 2).
- *tDurationDown*: The time duration between the peak (T_peak_) and the end (T_end_) of the T-wave (Figure 2).
- *stRise-perc*: It is calculated as (Amplitude (T_on_) − Amplitude (QRS_end_))/(Amplitude (T_peak_) − Amplitude (QRS_end_)).
- *stDuration*: The time duration of the ST segment is calculated as the time difference between QRS_end_ and the T_on_.
- *st-rrRatio*: The parameter is calculated as a ratio between the *stDuration* and the duration of the RR interval.

B. Features that are calculated from the area under the curve:

- *tAreaUpc*: The area under (in the case of positive T-wave) or above (in the case of negative T-wave) the ECG signal is time-limited by the beginning (T_on_) and peak (T_peak_) of the T-wave. The ECG amplitude values are shifted so that the T_on_ amplitude equals zero. The ECG values, which are lower than the amplitude of T_on_, are considered zero values in the case of a positive T-wave (or higher in the case of a negative T-wave). The integral is calculated using the rectangle method that starts at the peak point. The parameter assumes values greater than zero for positive T-waves and values lower than zero for negative T-waves.
- *tAreaDownc*: The area is calculated analogically as a *tAreaUpc*, just as the border values are used as T_peak_ and the end (T_end_) of the T-wave. The parameter also assumes values greater than zero for positive T-waves and values lower than zero for negative T-waves.
- *tAreac*: It is counted as a sum of the parameters *tAreaUpc* and *tAreaDownc* mentioned above.
- *tAreacUpDownRatio*: It is counted as the *tAreaUpc* divided by *tAreaDownc*.
- *stAreac*: The area under (in the case of a rising ST segment) or above (in the case of a decreasing ST segment) the time-limited ECG signal bordered by the QRS offset (QRS_end_) and the beginning of the T-wave (T_on_). The ECG amplitude values are shifted so that the QRS_end_ amplitude equals zero. ECG values lower than QRS_end_ amplitude are considered zero values. The integral is calculated using the rectangle method that starts at the peak point. The parameter assumes values greater than zero for the rising ST segments and values lower than zero for the decreasing ST.

C. Features based on the T-wave transformation to the unit circle:

- *oneAreaUp*: The T-wave is transformed to the unit circle, where the point of the ECG signal [Time(T_peak_), Amplitude(T_on_)] corresponds to the point [0,0] of the unit circle, [Time(T_peak_), Amplitude(T_peak_)] to the point [0,1], [Time(T_on_), Amplitude(T_on_)] to [−1,0]. Then, the area of the transformed T-wave is calculated analogously to *tAreaUpc*. The parameter’s value is always positive (Figure 2).
- *oneAreaDdown*: The T-wave is transformed into the unit circle, where the point of the ECG signal [Time(T_peak_), Amplitude(T_end_)] corresponds to the point [0,0] of the unit circle, [Time(T_peak_), Amplitude(T_peak_)] to the point [0,1], [Time(T_end_), Amplitude(T_end_)] to [1,0]. Then, the area of the transformed T-wave is calculated analogously to *tAreaDownc*. The value of the parameter is always positive (Figure 2).
- *ratioUpDown-perc*: The value is counted as the ratio *oneAreaUp*/*oneAreaDown*.
- *oneSTTAreaUp*: The T-wave is transformed into the unit circle, where the point of the ECG signal [Time(T_peak_), Amplitude(QRS_end_)] corresponds to the point [0,0] of the unit circle, [Time(T_peak_), Amplitude(T_peak_)] to the point [0,1], [Time(QRS_end_), and Amplitude(QRS_end_)] to [−1,0]. Then, the area of the transformed T-wave is calculated analogously to *tAreaUpc*. The parameter’s value is always positive.
- *ratioSTTUpDown-perc*: The value is counted as the ratio *oneSTTAreaUp*/*oneAreaDown*.

D. Combinations

- *tAreacPerSec*: The parameter is counted as a *tAreac*/*tDuration*. The ratio eliminates the influence of the variation in the RR interval.
- *tAreacUpPerSec*: The parameter is counted as a *tAreacUp*/*tDurationUp*. The ratio eliminates the influence of the variation in the RR interval.
- *tAreacDownPerSec*: The parameter is counted as *tAreacDown*/*tDuratioDown*. The ratio eliminates the influence of the variation in the RR interval.
- *stAreacPerSec*: The parameter is calculated as *stAreac*/*stDuration*.

Finally, the absolute values of all the features for the negative T-waves are used.

All the parameters mentioned above are calculated separately for each ECG beat of each signal. To suppress potential inaccuracies in the delineation, 20% of ECG cycles with the highest root mean square error are omitted as outliers. The values of these features are assumed to be consistent within a single signal. Subsequently, each signal is represented by the mean of the rest of the values for each parameter.

### 2.4. Statistical Analysis

The dataset exhibits a non-Gaussian distribution, characteristic of medical data where biological variability and measurement idiosyncrasies often preclude fitting to a normal distribution. Consequently, the statistical approach adopted herein is nonparametric, circumventing the need for the normality assumption and thus providing a more robust analysis given the data’s distributional properties.

#### 2.4.1. Discrimination Analysis

To effectuate the differentiation between the LQT3 genotype and its counterparts LQT1 and LQT2, the Wilcoxon rank sum test, also known as the Mann–Whitney U test, has been employed. This nonparametric test assesses the null hypothesis that two independent samples stem from the same distribution. Given our focus on the distinctiveness of the LQT3 group, this methodological choice allows for an assessment of the median differences between the groups without the constraints imposed by parametric tests. The significance threshold was set at 5%, aligning with conventional statistical practice to balance the risks of Type I and Type II errors.

#### 2.4.2. Correlation Analysis

To ascertain the independence of variables, we utilized the Spearman rank correlation coefficient—a nonparametric measure of correlation that assesses the strength and direction of the monotonic relationship between two continuous or ordinal variables. An absolute threshold of 0.6 for the Spearman coefficient was instituted to delineate correlated from uncorrelated features. This cut-off ensures the selection of features that provide unique information, minimizing redundancy in the predictive model.

#### 2.4.3. Feature Selection

A triad of mutually uncorrelated parameters that exhibited the most pronounced discrimination capability (see Discrimination Analysis) between LQT3 and the combined LQT1 and LQT2 cohorts was identified upon establishing correlation thresholds (see Correlation Analysis). These parameters were selected for their statistical significance and potential to contribute to a model that accurately classifies the genotypes based on ECG-derived attributes. The strategic selection of these variables is pivotal, as it directly influences the classifier’s ability to discern the subtle nuances across the genotype spectrum, thereby bolstering the predictive accuracy of the ensuing machine-learning model. Selecting only three parameters is a suitable compromise between the achievable accuracy of the classifier and the computational complexity.

### 2.5. Classification Model

Neural networks are highly effective and increasingly prevalent in medical applications. For instance, the study [21] demonstrates the successful application of Convolutional Neural Networks (CNN) on a large dataset of LQT1 and LQT2 patients. However, for this study, we found the SVM model to be a more suitable choice given the constraints of our smaller dataset. While neural networks are powerful, they are prone to overfitting and overtraining with limited data. Consequently, models such as LSTM (Long-Short-Term Memory) and CNN, which typically require extensive data for optimal performance, were not employed in this research.

SVM models, in contrast, offer several advantages for machine learning classification tasks. These include fast training times, high accuracy, and efficiency with smaller datasets and lower computational demands. They also maintain better transparency and explainability. In this study, the SVM parameters were optimized using a Gaussian kernel function [22]. Initially, the Box Constraints (C) and Kernel Scale (KS) were automatically optimized via MATLAB R2024b’s Classifier Learner Application. Subsequently, these parameters were further refined through two optimization methods: Grid Search, focused around the automatically generated values, and Bayesian optimization.

The three finest mutually uncorrelated parameters with the best discrimination of LQT3 are chosen as input variables of the classifier. Data vectors are weighted to suppress the influence of the different ranges of separated classes. The weights for the LQT3 patient group are set to 9.3 (counted as the number of patients from the training group who are carriers of LQT1 and LQT2 together divided by the number of LQT3 patients), with the rest at the value of weight one (Table 1).

#### Classifier Performance Measurements

All signals have been automatically split into the random non-stratified partitions (Table 1) of the training set and test set. The classifier is trained and cross-validated using only the training dataset (137 LQT1, 58 LQT2, 21 LQT3). The 5-fold cross-validation is chosen. The accuracy of the proposed classifier is then verified in the independent out-of-sample test dataset (68 LQT1, 28 LQT2, 10 LQT3). To determine accuracy, the test dataset is weighted 9.6 for the LQT3 group. This weight is calculated as the number of patients from the testing group who are carriers of LQT1 and LQT2 combined, divided by the number of LQT3 patients. The weights for the other genotypes in the testing dataset are set to one (Table 1).

To enhance the transparency and trustworthiness of the model, we applied the explainable artificial intelligence technique SHAP (Shapley Additive Explanations) [23] to our best performing model. SHAP enables the explanation and interpretation of decisions and predictions made by a machine learning model. By utilizing the entire dataset, SHAP provides a comprehensive global perspective on feature importance and interactions.

## 3. Results

This study presents a robust automated methodology for distinguishing the LQT3 genotype from the LQT1 and LQT2 genotypes using electrocardiogram (ECG) data. Our analysis encompassed 322 ECG recordings (Table 1), providing a substantial dataset for feature evaluation and genotype differentiation.

Our innovative approach identified three critical features that effectively differentiate LQT3, employing a novel, unbiased algorithmic process. These features include *tDurationUp*, *oneAreaUp*, and *stAreac*. The feature *tDurationUp*, a traditional temporal measure, quantifies the duration between the onset and peak of the T-wave, providing a straightforward temporal perspective of the ECG waveform.

Distinctly, *oneAreaUp* offers fresh insight by calculating the area under the ascending part of the T-wave after its transformation to the unit circle. This metric explicitly captures the convexity and concavity of the T-wave’s ascending segment, offering an innovative measure less affected by heart rhythm variations than traditional methods.

The third feature, *stAreac*, quantifies the area under the ST segment, adding another layer of diagnostic information by analyzing this specific part of the ECG curve. Statistical analysis of these features using the Wilcoxon rank sum test revealed highly significant *p*-values (less than 0.001 for *tDurationUp* and *oneAreaUp*, and 0.002 for *stAreac*), underscoring their discriminative power.

**Figure 3:**
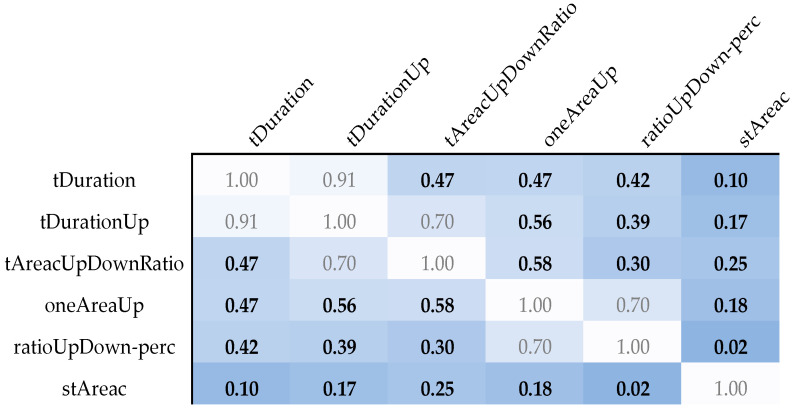
An overview of the Spearman correlation coefficients between parameters with a Wilcoxon rank sum test *p*-value lower than 0.005. The white color of the box corresponds to the maximum mutual correlation between parameters. The darker the color, the smaller the mutual correlation of the given pair of parameters.

**Figure 4:**
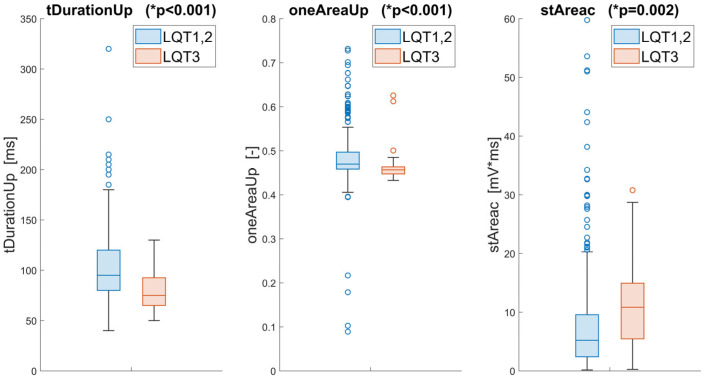
Statistical comparison and visualization of the three best features extracted from the ECG for the LQT3 and LQT1,2 groups. The * *p*-values of the Wilcoxon test are included for these parameters.

Additionally, the mutual independence of these features was confirmed via Spearman correlation coefficients, which were significantly below the critical threshold of 0.6, indicating minimal redundancy among the selected features (Figure 3). The distributions of these features across the different genotypes are visually depicted in Figure 4, illustrating the distinct patterns associated with LQT3 versus LQT1 and LQT2.

Together, these findings validate the effectiveness of the proposed features in genotype differentiation and highlight the potential of geometric transformations of ECG waves in enhancing diagnostic accuracy in the clinical genetics of long QT syndrome.

To differentiate between the LQT3 genotype and its counterparts using a feature set derived from electrocardiogram data, we employed a binary support vector machine (SVM) model with a Gaussian kernel function. The hyperparameters Box Constraints (C = 1) and Kernel Scale (KS = 1.3) were optimized using a five-fold cross-validation procedure in the MATLAB Classifier Learner Application. The suitability of the chosen SVM model was validated in this application by comparing its classification performance with that of other predefined models. These results, however, go beyond the scope of this study and are therefore not included here.

**Figure 5:**
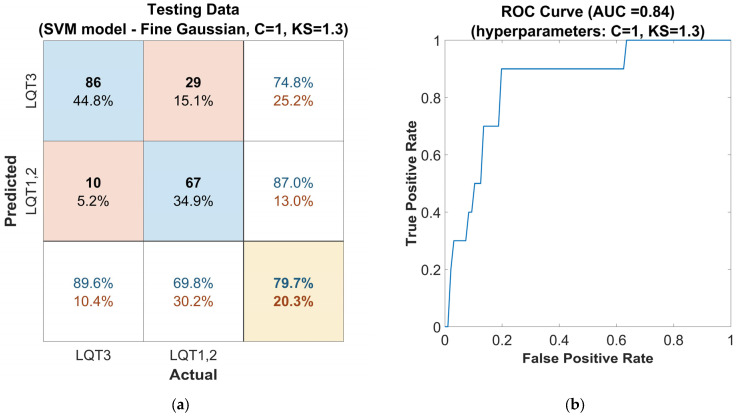
Performance of the SVM classifier model tuned with the Gaussian kernel function on the out-of-sample testing dataset, with automatically optimized hyperparameters Box Constraints (C = 1) and Kernel Scale (KS = 1.3): (**a**) confusion matrix for the classifier. The rows of the matrix correspond to the predicted class, and the columns correspond to the ground true class. Each cell includes the number of observations and the percentage of the total number of observations. The column on the far right of the plot shows the precision (blue) and false discovery rate (red). The row at the bottom of the plot shows the recall (blue) and false negative rate (red). The cell in the bottom right of the plot shows the overall accuracy. (**b**) ROC curve of the proposed classifier. The blue line represents the detection of LQT3.

The further evaluation involved applying the optimized classifier to an independent out-of-sample dataset (Figure 5a), where it maintained an accuracy of 80%, a sensitivity of 90%, and a precision of 75% for LQT3 discrimination. The proposed SVM model achieved an F1-score of 0.82. Figure 5b presents the receiver operating characteristic (ROC) curve of this model with the weighted area under the ROC curve (AUC) 0.84.

**Figure 6:**
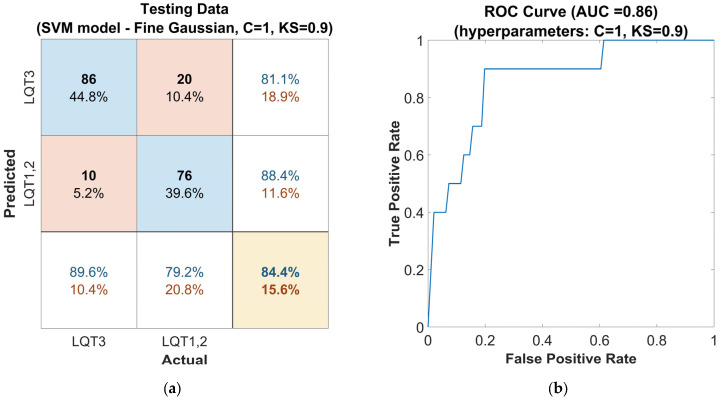
Performance of the SVM classifier model tuned with the Gaussian kernel function on the out-of-sample testing dataset, with hyperparameters Box Constraints (C = 1) and Kernel Scale (KS = 0.9) optimized using the Grid Search method: (**a**) confusion matrix for the classifier. The rows of the matrix correspond to the predicted class, and the columns correspond to the ground true class. Each cell includes the number of observations and the percentage of the total number of observations. The column on the far right of the plot shows the precision (blue) and false discovery rate (red). The row at the bottom of the plot shows the recall (blue) and false negative rate (red). The cell in the bottom right of the plot shows the overall accuracy. (**b**) ROC curve of the proposed classifier. The blue line represents the detection of LQT3.

Subsequently, the hyperparameters of the SVM model were further optimized using the Grid Search method, which was applied in the vicinity of the hyperparameters automatically optimized by the Classifier Learner Application, as mentioned in the previous step. The hyperparameters were optimized to the values of C = 1 and KS = 0.9. The evaluation on the out-of-sample dataset for this model configuration (Figure 6a) shows a solid accuracy of 84%, a high sensitivity of 90%, and a precision of 81% for LQT3 discrimination. The SVM model with these settings achieved an F1-score of 0.85 and an AUC of 0.86 (Figure 6b). This performance attests to the classifier’s capability to handle new, unseen data, which is crucial for practical clinical applications.

**Figure 7:**
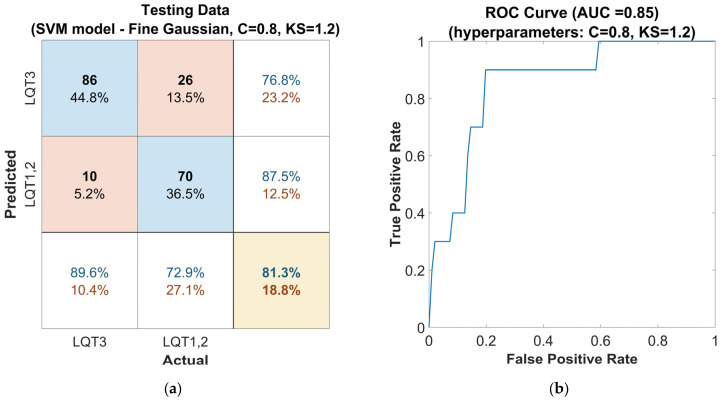
Performance of the SVM classifier model tuned with the Gaussian kernel function on the out-of-sample testing dataset, with Bayesian optimized hyperparameters, the Box Constraints (C = 0.8), and Kernel Scale (KS = 1.2): (**a**) confusion matrix for the classifier. The rows of the matrix correspond to the predicted class, and the columns correspond to the ground true class. Each cell includes the number of observations and the percentage of the total number of observations. The column on the far right of the plot shows the precision (blue) and false discovery rate (red). The row at the bottom of the plot shows the recall (blue) and false negative rate (red). The cell in the bottom right of the plot shows the overall accuracy. (**b**) ROC curve of the proposed classifier. The blue line represents the detection of LQT3.

Finally, the second approach, Bayesian optimization of hyperparameters, was applied. With the parameters set to C = 0.8 and KS = 1.2, the model achieved an accuracy (Figure 7a) of 81%, a sensitivity of 90%, and a precision of 77% for LQT3 discrimination. The F1-score was 0.83. This model configuration is presented with a ROC curve in Figure 7b showing an AUC of 0.85.

The highest performance was achieved using the classifier (Figure 6) optimized by the Grid Search method. To provide a global explanation of the final model, the SHAP method was applied using the entire dataset. Shapley values assign an importance value to each feature, representing its contribution to the model’s predictions. These values illustrate how each feature influences the final predictions, the relative significance of each feature, and the model’s dependence on interactions between features. The mean Shapley values for each parameter are shown in Table 2.

**Figure 8:**
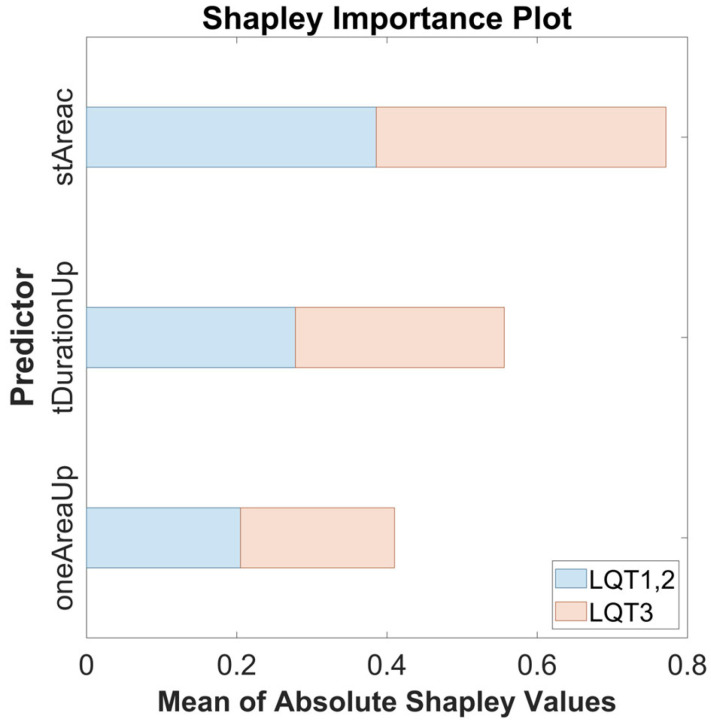
The Shapley importance plot showing the mean of absolute Shapley values for the features *stAreac*, *tDurationUp*, and *oneAreaUp* across both predicted groups: LQT3 (red) and LQT1,2 (blue).

The calculated values are graphically demonstrated in the following figure (Figure 8). The average absolute Shapley values are consistent across both classes, indicating that the importance of each feature is stable across both groups. *stAreac* is the most important feature, followed by *tDurationUp* and *oneAreaUp*. All features are significant.

These results underscore the final model’s high potential in distinguishing the LQT3 genotype from others. Using the three selected features has endowed the classifier with significant discriminatory power, affirming the utility of these features in genetic diagnostics related to long QT syndrome. This combination of high sensitivity and moderate precision paves the way for further refinement, aiming at an optimal balance that could enhance clinical decision-making in cardiovascular genetics.

## 4. Discussion

Cardiovascular problems, in general, are a significant topic to solve. Long QT syndrome belongs to those diseases that can lead to dangerous ventricular arrhythmias and may end with sudden cardiac death. Further research and improvement of its diagnostics can save many human lives. In our study, attention is paid primarily to the discrimination of patients with the LQT3 genotype. We consider this group of patients the most at risk because the effectiveness of beta-blocker treatment for them is debatable, and activities triggered by sleeping and resting are challenging to prevent. Very well-interpretable parameters were created to describe this genotype group. In this work, two novel approaches are presented. The parameters connected with the area under the curve represent a combination of amplitude and the duration of the analyzed part of the signal. The attitude of the transformation ECG curve to the unit circle gives information about the wave part’s shape (convexity and concavity), regardless of its duration. Approaches were proposed, among others, to suppress the influence of heart rhythm. An interesting idea for further research could be calculating the sigmoid parameters [24] after transforming the ECG curve into a unit circle. Although the parameters are primarily designed to differentiate the LQT3 group, further research should aim to distinguish between all three main genotypic groups.

Although sudden pathological situations are life-threatening, their underlying source is genetically caused channelopathies, the presence of which is constant and long-lasting. Therefore, we assumed that the identification of permanent changes in ECG signals can be detected even in short resting sections of the ECG, which can be part of regular preventive cardiology examinations. These changes could also probably be identified from ECG signals measured by smartwatch apps. For this case, the measurement of Lead I must be sufficient. To test the broadest possible applicability of this method for the future, only the first channel of the available signals was used for this work.

As mutually uncorrelated parameters with the best ability to differentiate the LQT3 group were selected, the time duration of the ascending part of the T-wave (*tDurationUp*), the area of the ascending part of T-wave after the transformation to the unit circle (*oneAreaUp*), and the area under the ST segment (*stAreac*). These three features, in combination with the use of SVM tuned by the optimized Gaussian kernel function (C = 1, KS = 0.9), can distinguish the LQT3 genotype group of patients with an accuracy of 84%, sensitivity of 90%, precision of 84%, and F1-score of 0.85. The AUC for the proposed model is 0.86. The results achieved in our study are better, especially in the precision value, than those published by Bos [11]. In Bos’s study, the results were presented with an accuracy of 80%, sensitivity of 78%, precision of 33%, F1-score of 0.46, and AUC of 0.86.

The advantage of our study is that we did not use neural networks. Compared to neural networks, the SVM model can be very effective even when trained on a smaller amount of data, can have lower computational demands, and is interpretable in a more transparent way, which is crucial for healthcare applications. In addition, our classification based on only Lead I of the ECG signal with a sampling frequency of only 200 Hz provides broader possibilities of utilization even for more straightforward applications.

An 84% classification accuracy might not be considered optimal; however, it is important to recognize that the accuracy of detection from ECG signals likely has its inherent limitations. This can be attributed to overlaps in certain ECG patterns among patients with the LQT1 and LQT3 genotypes. For example, the study [15] quantified this overlap at 33%. Similarly, the study by Bos [11], focusing on genotype discrimination in LQTS, demonstrates that 31% of patients with the LQT3 genotype are incorrectly identified as LQT1, while 6% of patients with LQT1 are misclassified as LQT3. Based on these findings, it can be inferred that in some cases, we may be approaching the boundaries of distinguishability.

Future work building on this research should focus on achieving a more comprehensive differentiation among the three main genotypic groups of LQTS. In further studies, the proposed parameters should also be tested on a more extensive genotyped database, ideally utilizing a 12-lead ECG. Information from multiple input channels could enable a more detailed analysis, provide additional input parameters, and potentially lead to the development of more robust models with high diagnostic value. Additionally, if a significantly larger dataset becomes available, it will be possible to employ other machine learning methods, including neural networks, to further enhance the analysis.

### Limitations—Reflective Considerations on Methodological Constraints

The innovative approach employed in our study for the automatic extraction of resting segments from extensive Holter ECG recordings has demonstrated significant utility, setting a precedent for analyzing vast electrophysiological databases. Nevertheless, this technique is not without its constraints, which warrant a candid discussion.

Firstly, while efficient, the automatic selection algorithm may occasionally fall short of achieving the precision that manual segmentation offers. The inherently complex and variable nature of cardiac signals means that automated processes might inadvertently overlook nuanced features, potentially impacting the fidelity of the extracted data. Although manual correction could enhance the dataset’s quality, such an undertaking would be labor-intensive and less feasible on a larger scale. The balance between automation and accuracy is, therefore, a delicate one, with implications for the scalability of our methods. Likewise, adding additional input parameters to the model can increase classification accuracy, but at the cost of greater computational complexity.

Secondly, while using open-source delineation tools facilitates widespread accessibility and ease of use, it introduces limitations, particularly in cases with atypical ECG morphologies such as flat or notched T waves. In such instances, the precision of automatic delineation may be compromised, suggesting a need for specialized algorithms or manual oversight to capture these aberrant waveforms accurately.

Furthermore, the representativeness of the dataset, particularly concerning the LQT3 genotype, poses a limitation. The relative paucity of LQT3 patient data compared to other genotypes may impede the model’s ability to learn the distinctive features of this subgroup, thus potentially affecting the generalizability of our findings across all manifestations of LQTS. Augmenting the dataset with a larger spectrum of LQT3 cases could enhance the robustness of the model.

Notwithstanding these limitations, the breadth and depth of the THEW database should not be understated. As one of the most expansive collections of genotyped LQTS data, it is a vital resource for the field. The continuous expansion of such databases and the advent of automated diagnostic tools in routine clinical screenings hold great promise. These advancements may lead to the early detection of LQTS, thus offering a proactive avenue for managing this condition and contributing valuable insights for future research.

In summary, while acknowledging our study’s methodological constraints, it is imperative to recognize the potential of these automated tools in transforming the landscape of LQTS diagnosis and management. The iterative refinement of these methodologies, informed by the limitations noted, will undoubtedly enhance the precision and applicability of machine learning in cardiac electrophysiology.

## 5. Conclusions

Our study demonstrates that an SVM classification model can effectively distinguish the LQT3 genotype from LQT1 and LQT2 using single-lead ECG data at 200 Hz. We introduced two parameterization techniques—area under the ECG curve and transformation into a unit circle—which were instrumental in identifying LQTS genotypes. While promising for cardiological diagnostics, the applicability of these methods to other cardiac conditions requires further investigation. Expanding the genotype range and patient cohort in future research could validate and enhance these findings. This study highlights the potential of machine learning to streamline non-invasive LQTS diagnoses, paving the way for broader clinical applications.

## Figures and Tables

**Table 1:** Provides an overview of the actual and balanced numbers of signals, along with the appropriate weights in the training and testing datasets, depending on the LQTS genotype.

	Training Dataset			Testing Dataset		
LQTS Groups	LQT1	LQT2	LQT3	LQT1	LQT2	LQT3
Number of signals	137	58	21	68	28	10
Weights	1	1	9.3	1	1	9.6
Balanced number of signals	137	58	195	68	28	96

**Table 2:** Provides an overview of mean Shapley values for all input features of each predicted group.

Predictor	LQT1,2	LQT3
*tDurationUp*	0.29	0.29
*oneAreaUp*	0.22	0.22
*stAreac*	0.41	0.41

## Data Availability

The dataset used in this study is provided by Holter ECG Warehouse (THEW). Specifically, we utilized the genotyped long QT syndrome subset E-HOL-03-0480-013 [16].

## Abbreviations

The following abbreviations are used in this manuscript:

AUC	Area under the receiver operating characteristic curve
C	Box Constraints
CNN	Convolutional Neural Networks
ECG	Electrocardiogram
HF	High frequency
HRV	Heart rate variability
LF	Low frequency
LQTS	Long QT syndrome
LSTM	Long-Short-Term Memory
KS	Kernel Scale
ROC	Receiver operating characteristic
SHAP	Shapley Additive Explanations
SVM	Support Vector Machine
TdP	Torsades de pointes
THEW	Telemetric and Holter ECG Warehouse
